# An *Ex Vivo* Model for Studying Hepatic Schistosomiasis and the Effect of Released Protein from Dying Eggs

**DOI:** 10.1371/journal.pntd.0003760

**Published:** 2015-05-12

**Authors:** Geoffrey N. Gobert, Sujeevi K. Nawaratna, Marina Harvie, Grant A. Ramm, Donald P. McManus

**Affiliations:** QIMR Berghofer Medical Research Institute, Herston, Queensland, Australia; McGill University, CANADA

## Abstract

**Background:**

We report the use of an *ex vivo* precision cut liver slice (PCLS) mouse model for studying hepatic schistosomiasis. In this system, liver tissue is unfixed, unfrozen, and alive for maintenance in culture and subsequent molecular analysis.

**Methods and Findings:**

Using thick naive mouse liver tissue and sterile culture conditions, the addition of soluble egg antigen (SEA) derived from *Schistosoma japonicum* eggs, followed 4, 24 and 48hrs time points. Tissue was collected for transcriptional analysis and supernatants collected to quantitate liver enzymes, cytokines and chemokines. No significant hepatotoxicity was demonstrated by supernatant liver enzymes due to the presence of SEA. A proinflammatory response was observed both at the transcriptional level and at the protein level by cytokine and chemokine bead assay. Key genes observed elevated transcription in response to the addition of SEA included: IL1-α and IL1-β, IL6, all associated with inflammation. The recruitment of antigen presenting cells was reflected in increases in transcription of CD40, CCL4 and CSF1. Indications of tissue remodeling were seen in elevated gene expression of various Matrix MetalloProteinases (MMP3, 9, 10, 13) and delayed increases in TIMP1. Collagen deposition was significantly reduced in the presence of SEA as shown in COL1A1 expression by qPCR after 24hrs culture. Cytokine and chemokine analysis of the culture supernatants confirmed the elevation of proteins including IL6, CCL3, CCL4 and CXCL5.

**Conclusions:**

This *ex vivo* model system for the synchronised delivery of parasite antigen to liver tissue provides an insight into the early phase of hepatic schistosomiasis, corresponding with the release of soluble proteins from dying schistosome eggs.

## Author Summary

Schistosomiasis is a major disease of humans caused by schistosome blood flukes. One of the primary sites of pathology is the liver of the infected host, and it is caused by the deposition of parasite eggs leading to an immune-fibrotic response. We have used an animal tissue model for this disease, where slices of liver of uniform thickness are used in culturing experiments with the addition of parasite egg protein from the species *Schistosoma japonicum*. We found over the 48 hours tissue was exposed to egg proteins, gene associated with important immunological responses including inflammation and antigen processing were up-regulated. Similarly, responses in tissue remodeling and the modulation of genes involved in fibrosis were also indicated. In this study we were able to characterise the initial responses of host liver tissue when exposed to parasite protein released by dying eggs, representing one of the early phases of hepatic schistosomiasis in an *ex vivo* model.

## Introduction

The major neglected tropical disease schistosomiasis is caused by parasitic helminth worms of the genus *Schistosoma*. The disease affects 200 million people worldwide with many more at risk. The clinical features of schistosomiasis range from fever, headache and lethargy, to severe liver inflammation, liver scarring (collagen deposition and fibrosis), and organ failure. Within host tissues containing trapped schistosome eggs, extensive fibrosis occurs but, paradoxically, the immediate area surrounding the eggs themselves remains unaffected [1–3].

The granulomatous reaction and general immune reaction induced by schistosome egg trapped within host tissues, is the primary etiology of schistosomiasis [4]. While granulomas arise to destroy the eggs and sequester or neutralise otherwise pathogenic egg antigens, this process also leads to extensive fibrosis of host tissues [5]. Schistosome eggs can survive at most for a few weeks in host tissue [6], after which their soluble protein contents are released. The majority of pathology resulting from *S*. *mansoni* or *S*. *japonicum* infections develops at the site of maximal egg accumulation: the liver [7]. In schistosomiasis, host liver pathology is induced by a CD4+Th2-driven granulomatous response directed against schistosome eggs and the molecules they release when lodged in host tissues. The Th2 cytokines IL-4 and IL-13 drive this response, while IL-10, IL13R-α2, IFN-γ, and a subset of regulatory T-cells and alternatively activated macrophages, act to limit the schistosome-induced pathology. The majority of these observations were made using mouse models for schistosomiasis with egg deposition in the liver staggered over weeks. Coordinated egg deposition is possible using the pulmonary granuloma model [8], which also controls the amount of antigen introduced to the animal but not the distribution of antigen across the organ, which in this case is obviously the lung, not the normal site of pathology, the liver. We have adopted the precision cut liver slice technique [9] which provides an *ex vivo* system that allows the consistent concentration of parasite antigen across the entire tissue section in a synchronized manner and permits the collection of tissue through a time course.

The Hepatic Stellate Cell (HSC) is normally a quiescent non-parenchymal cell located in the space of Dissé of the liver sinusoid. Upon insult or injury to the liver, HSCs undergo a process of transdifferentiation (activation) into a fibrogenic myofibroblast responsible for the production of collagen and the formation of scar-like matrix (fibrosis). The HSC plays a significant role in fibrotic schistosomiasis, as shown in both murine and human infections of *S*. *japonicum* [10] and human *S*. *mansoni* infections [11].

In recent studies we and others have co-cultured HSCs with schistosome eggs (separated by transwells), and measured known markers of transdifferentiation, which demonstrated that cells remained in, or were returned to, a quiescent state [12,13]. Even in the presence of TGF-β, the down-regulation of fibrosis-related genes, αSMA and procollagen α1(I), and the up-regulation of PPARγ, which is associated with quiescence, was observed in HSCs [12]. TGF-β is a strong inducer of profibrotic genes resulting in HSC activation. The ability of schistosome eggs to attenuate this strong profibrotic effect of TGF-β is a significant observation and supports the hypothesis that an egg molecular component could be used to treat active fibrotic disease. This down-regulation of fibrosis associated genes could explain why collagen deposition is observed only at the periphery of the granuloma distal to the location of the eggs and why fibrosis only occurs throughout the granuloma area after an egg has been destroyed. While recent work by others using primary HSCs and *S*. *japonicum* soluble egg antigen (SEA) reported an increase in cell activation [14], this study did not control for the presence of LPS (lipopolysaccharide) and used SEA concentrations 20–50 times higher than we have employed [13]; moreover a second research group has now confirmed our anti-fibrotic phenotype in primary HSCs using LPS-free *S*. *japonicum* SEA [15].

To further characterise this phenotype we have used SEA with an *ex vivo* precision cut liver slice (PCLS) mouse model, and whole genome transcriptomics. The use of SEA is relevant as it replicates the released proteins from dying schistosome eggs and is a protein mixture distinct from excreted/secreted (ES) products actively released from live eggs. The tissue slice culture model of naïve murine liver allows the investigation of local responses of a mixture of hepatic cells (HSCs, hepatocytes and Kupffer cells (KCs)) to egg bioactive molecules, better replicating the *in vivo* liver microenvironment, and determining how early molecular events may contribute to the eventual granulomatous response. Similar approaches using PCLS have been successfully employed in studies of liver metabolism and toxicity [16,17]. Fibrosis has also been investigated using tissue slices of liver, predominantly using chemical or surgical methods (see [18] for review). However the application of *ex vivo* model to investigate innate immunity and tissue remodeling in the liver, in response to a major human pathogen, is a novel approach.

## Materials and Methods

### Ethics statement

All work was conducted with the approval of the Animal Ethics Committee of the QIMR Berghofer Medical Research Institute (Project Number 288), which adheres to the Australian code of practice for the care and use of animals for scientific purposes, as well as the Queensland Animal Care and Protection Act 2001; Queensland Animal Care and Protection Regulation 2002.

### Parasite lifecycle and antigen collection

The *S*. *japonicum* life cycle is currently maintained at QIMR Berghofer in female Swiss mice infected with a Chinese mainland, Anhui population, strain. Live schistosome eggs were isolated from the livers of murine hosts as previously described [12]. Isolated *S*. *japonicum* eggs were homogenised (twice using 5 mm beads) using tissue lyser (Qiagen) for 1 min each at 25 Hertz. The subsequent homogenate was checked microscopically to ensure the complete rupturing of eggs. The homogenate was then centrifuged 10,000 g for 2hrs at 4°C and the supernatant collected. All egg antigen preparations (SEA) were shown to be lipopolysaccharide (LPS)-free by the Limulus Amebocyte Lysate (0.06 EU/ml) assay (Lonza, Australia).

Mice (naïve female C57BL/6, 5–6 weeks old) were euthanised by cervical dislocation and intact livers removed. A portion of liver was collected at this point and was designated pre-cut [PC]. An additional ~1cm^3^ piece of liver was collected and a refrigerated macro tissue slicer (WPI model SYS-NVSLM1 Motorized Vibroslice) was used to cut 250 μm sections of mouse liver (slices), which were then cultured in William’s Medium E containing 25 mM glucose, 10 mg/ml gentamycin and 10% (v/v) FBS [16], for 2 hours to allow the tissue to rest. Then, SEA was added to the cultured hepatic tissue slices at 10 μg/ml (concentration based on [12] and [2,19]) for a total of 48hrs; negative controls included no added SEA protein. The SEA concentration used was also indicative of the amount of SEA we isolate from an infected mouse liver, and the tissue volume the granuloma is contained within the organ. Tissue sections were then collected after 0 (prior to SEA addition), 4, 24 and 48hrs and tissue processed for transcriptional analyses. Four biological replicates (liver slices from 4 separate mice) were examined for all of the methods listed below.

### Hepatotoxicity assay

Hepatotoxicity was determined by assessing lipid peroxidation using a malondialdehyde (MDA) Assay Kit (BioVision, San Francisco USA) using the media sampled at each time point [20]. Quantification of MDA is reflective of oxidative stress, with the formation of MDA being one of the by-products of lipid peroxidation [20]. The absorbance at 532nm was measured after the formation of MDA-TAA (Thiobarbituric Acid) adducts using the manufacturer’s standard protocol.

### Microarray analysis: cRNA synthesis and whole genome microarray hybridisation

cRNA was synthesised from total RNA for each sample using Illumina Total Prep RNA amplication kits (Ambion). Biotin-labeled cRNA was hybridised to Illumina Mouse Ref-8 Version 2 whole genome expression arrays, which were then scanned using an Illumina BeadStation (Illumina).

### Microarray analysis: Feature extraction and data analysis

Quality control of microarray data involved the checking of signal-intensity histograms for hybridisation efficiency and experimental noise using GenomeStudio (Illumina), represented as a detection score. Further quality control is termed “Flags” and is calculated on a gene specific basis; it reflects the internal quality controls that are used by microarray manufacturers, considering both the hybridisation and scanning phases, and the overall fidelity of the extracted data. All subsequent analysis was performed using GeneSpring GX Version 12.5, and expression data were normalised to the median of all samples. Gene lists were first normalised to uninfected controls and filtered for Flags and signal significance based on the detection score of d ≥ 0.95 (which equates to a confidence value of P≤0.05). All gene expression data are publicly available through NCBI’s Gene Expression Omnibus; Series Accession Number: GSE64071 and Platform Number: GPL6885.

One-way ANOVA with Benjamini-Hochberg correction for multiple testing was then used to identify genes differentially expressed (P≤0.05) over the entire time-course [2]. Alternatively, a moderated t-test with multiple testing correction p≤0.05 (Benjamin-Hochberg) was used to identify differentiated expression based on the presence of *S*. *japonicum* SEA. The filtered microarray data provided the basis for ranking genes derived from fold changes and significance values. Ingenuity pathway analysis was used to identify diseases and biofunctions or canonical pathways that were over-represented by the differentially expressed genes [21]. Network analysis (IPA- Ingenuity Pathway Analysis) was performed to create an overview of the main signalling processes occurring in the SEA exposed liver slices. Up-stream regulator analysis was performed to identify common upstream factors (e.g., transcription factors or drugs) which could be regulating the observed response. Fold changes were represented as treated (with SEA) relative to untreated samples and p-values associated with these fold changes calculated from non-averaged time point and treatment groups. A moderated T-test with no-multiple testing correction was employed to statistically compare fold changes between treated and untreated samples at each time point (since n = 4 for biological replicates).

### Real-time PCR

cDNA was synthesised from total RNA using a Quantitect reverse transcription kit (Qiagen), and cDNA concentrations were quantified using a Nanodrop-1000 spectrophotometer. Real-time PCR was performed to validate a subset of microarray data. Primers for quantitative RT-PCR were sourced from the literature [22–26] or designed using Primer 3 software (S1 Table). Real-time data were normalised to the housekeeping gene, hypoxanthine phosphoribosyltranferase (HPRT), as reported previously [27]. Real-time PCR was performed using SYBR Green master mix (Applied Biosystems) on a Corbett Rotor Gene 6000 (Corbett Life Sciences). Data from the microarray and real time PCR analyses were examined to ascertain if they fitted normal distributions using the D'Agostino and Pearson omnibus and the Shapiro-Wilk normality tests. Statistical analyses were conducted using GraphPad Prism V6.05 or Microsoft Excel.

### Analysis of culture supernatant

Cytokine and chemokine levels were detected from culture supernatant using the Legendplex Mouse Proinflammatory Chemokine Capture Beads and Mouse Th cytokine Capture Beads multi-analyte flow assay kits (Biolegend) as per manufacturer’s instructions. The Chemokine Capture Beads measured CCL2, CCL3, CCL4, CCL5, CCL11, CCL17, CCL20, CXCL1, CXCL5, CCL22, CXCL9, CXCL10 and CXCL13. The Th Cytokine Capture Beads measured IL-2, IL-4, IL-5, IL-6, IL-9, IL-10, IL-13, IL-17A, IL-17F, IL-21, IL-22, IFN-γ and TNF-α. Data were collected on a Fortessa five laser flow cytometer (Becton Dickinson), and analysed using FCAP Array software (Becton Dickinson).

## Results

### MDA assay

**Fig 1:**
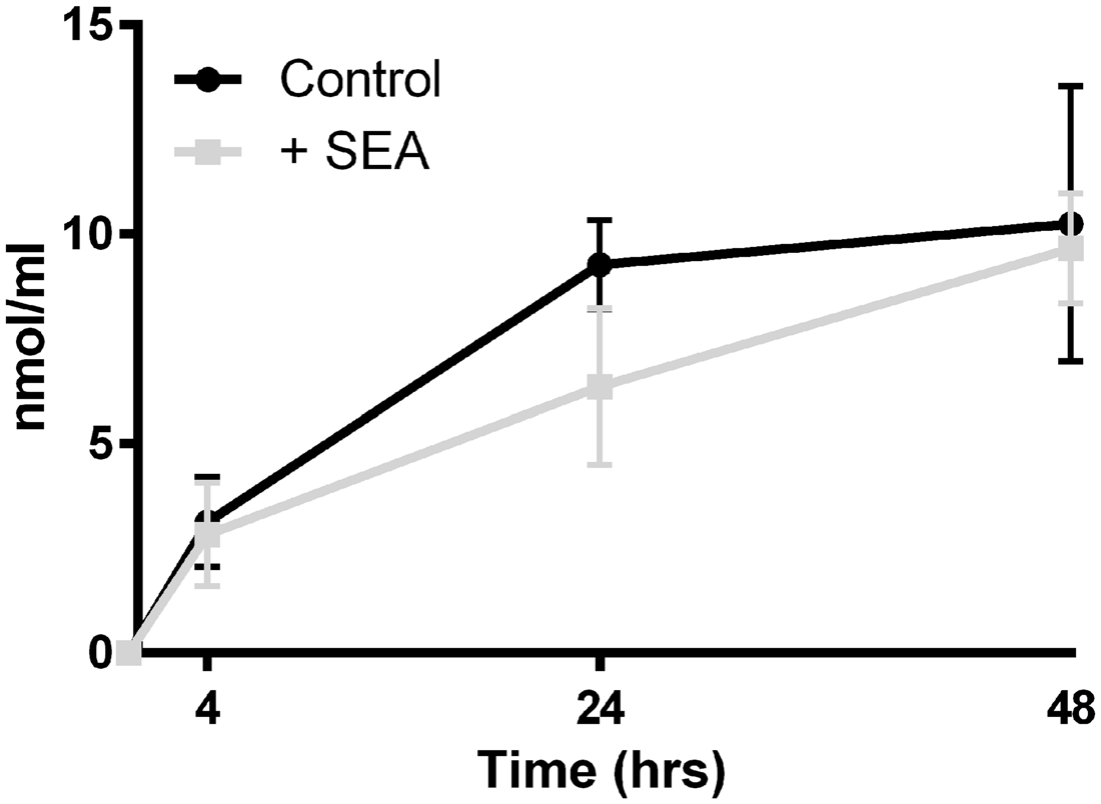
Malondialdehyde (MDA) assay of culture media containing liver slice tissue exposed or not to soluble egg antigen (SEA). While MDA concentrations in media increased over the 48 hour time course, indicating hepatotoxicity, there was no statistical difference between treatment groups at any time point (p-value>0.05).

There was an increase in the MDA content of the media of cultured liver slices over the course of the observed 48hrs; however the addition of SEA did not result in greater hepatotoxicity when compared with controls (P value >0.05) (Fig 1).

### Transcriptional findings

**Fig 2:**
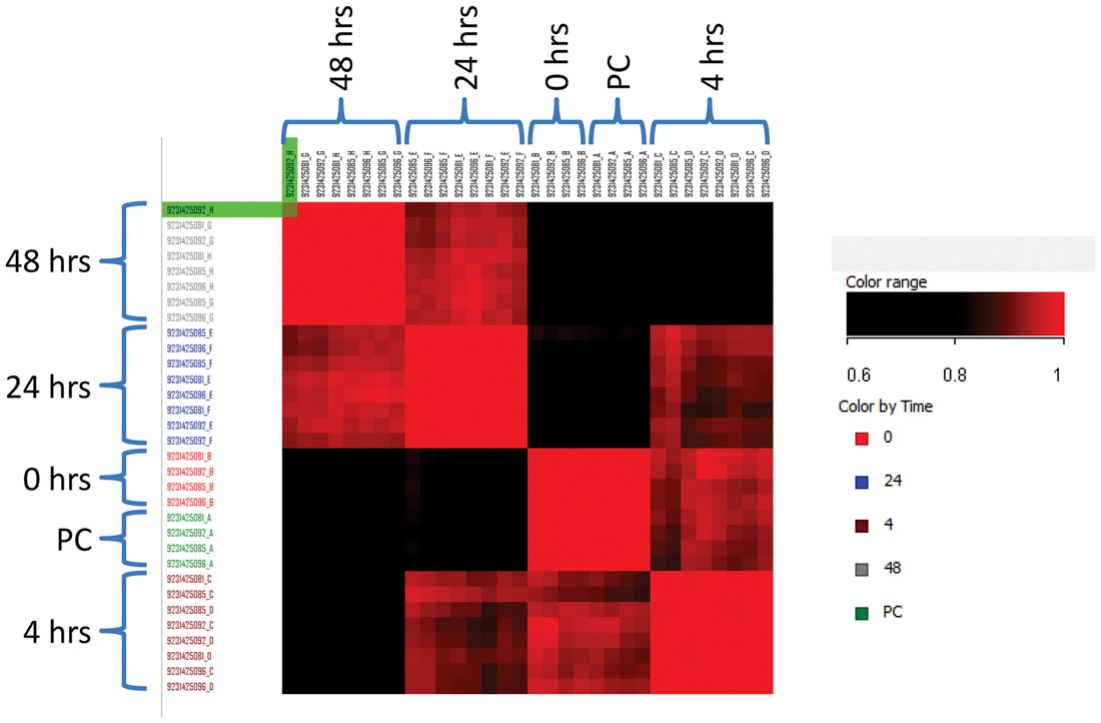
Correlation of 13,746 genes identified after quality control filtering. Colouring ranging from r^2^ = 0.6 for black to r^2^ = 1 red. Individual samples are grouped by time point and correlation between biological replicates varied between r^2^ = 0.965–0.985. Correlation between individual time points with pre-cut (whole) tissue resulted in r^2^ = 0.97 for time point 0 (after cutting and 2 hours rest), 0.88 after 4 hours, 0.73 after 24 hours and 0.57 after 48 hours.

Filtering of microarray data started with the complete 48,803 probes which were then filtered for flags (≥8/32 samples) reducing the number to 22,902. We then applied filters for detection score (d≥0.95 equivalent of p-value ≤0.05, ≥8/32 samples) reducing the list to 13,746 probes (S2 Table). Correlation between individual samples using the 13,746 probes within biological replicates demonstrated a low level of variation r^2^ = 0.965–0.985 (Fig 2). Correlation between individual time points when compared to pre-cut tissue, resulted in r^2^ = 0.97 for time point 0 (after cutting and 2 hours rest), 0.88 after 4 hours, 0.73 after 24 hours and 0.57 after 48 hours. This indicates the degree that gene expression of tissue sliced liver varied from that of whole intact tissue, over the 48 hours in culture. Differential expression due to the time course culturing conditions, using 13,746 probes and 1-way ANOVA with multiple testing correction p≤0.05 (Benjamin-Hochberg), found that the majority of genes were affected as seen in 12,068 probes (S2 Table Column L). No further analysis of this gene list was undertaken.

A moderated t-test with multiple testing correction p≤0.05 (Benjamin-Hochberg) was used to identify differential expression based on the presence of SEA, i.e. treated versus untreated, reducing 13,746 probes to 48 individual genes (Table 1). While 1-way ANOVA based on time retained the majority of genes, indicating that most genes were differentially expressed due to being in culture, a much smaller number of genes were changed due to the presence of SEA. Of the 48 differentially expressed genes attributed to the presence of SEA, the largest increases in fold change across 4|24|48 hours were seen in IL-1 (interleukin-1- α and β, 2.4|3.3|4.3 fold and 5.2|4.1|10.7 fold); and FPR2 (formyl peptide receptor 2, 2.7|3.5|6.8 fold). Chemokines CXCL2 and CCL3 were also up-regulated (chemokine C-X-C motif ligand 2, 2.5|1.4|2.8 fold; chemokine C-C motif ligand 3, 2.8|2.3|2.4 fold). Only 2 genes were shown to be down-regulated over the time course due to the presence of SEA, this included PLD4 and LPL (phospholipase D 4, 1.1|1.3|-2.0 fold and lipoprotein lipase 1.5|-2.5|-5.2 fold).

**Table 1:** All 48 genes differentially expressed in response to *S*. *japonicum* soluble egg antigen (SEA).

	FC SEA / control (hours)						
Symbol	4	p-Value	24	p-Value	48	p-Value	Definition
il1b	5.2	7.66E-09	4.1	4.15E-09	10.7	8.41E-18	interleukin-1- beta
fpr2	2.7	6.91E-06	3.5	7.53E-07	6.8	4.21E-15	formyl peptide receptor 2
il1a	2.4	1.16E-05	3.3	2.48E-06	4.3	2.73E-11	interleukin-1-alpha
saa3	1.1	3.61E-01	1.9	4.94E-02	3.6	2.69E-08	serum amyloid A 3
cxcl2	2.5	2.52E-04	1.4	3.88E-04	2.8	3.68E-09	chemokine (C-X-C motif) ligand 2
irak3	1.2	8.93E-03	1.8	9.76E-05	2.5	3.64E-08	interleukin-1 receptor-associated kinase 3
ccl3	2.8	5.90E-06	2.3	3.50E-09	2.4	1.46E-08	chemokine (C-C motif) ligand 3
s100a8	1.0	2.54E-01	2.5	6.33E-06	2.3	4.83E-09	S100 calcium binding protein A8
ms4a7	1.1	6.46E-02	1.3	2.38E-03	2.0	9.47E-08	membrane-spanning 4-domains, subfamily A, member 7
fpr1	1.0	3.89E-01	1.5	1.98E-04	1.8	3.85E-06	formyl peptide receptor 1
tlr2	1.3	1.41E-02	1.3	9.44E-04	1.8	4.91E-06	toll-like receptor 2
ccl4	2.0	2.27E-02	1.5	3.98E-03	1.6	5.95E-04	chemokine (C-C motif) ligand 4
h2-m2	1.0	7.29E-01	1.7	3.32E-05	1.6	3.03E-05	histocompatibility 2, M region locus 2
cd207	1.7	8.02E-06	1.3	1.99E-03	1.5	5.36E-03	CD207 antigen
fyb	1.0	7.36E-01	1.5	5.59E-05	1.5	3.64E-04	FYN binding protein
clecsf9	1.5	4.26E-05	2.0	2.86E-06	1.4	1.16E-03	C-type lectin domain superfamily member 9
e330036i19rik	1.2	3.52E-02	1.2	3.40E-03	1.4	5.26E-04	RIKEN cDNA E330036I19 gene
pla2g15	1.0	5.21E-01	1.4	4.24E-05	1.4	3.27E-04	phospholipase A2, group XV
tiam1	1.0	3.87E-01	1.1	1.98E-01	1.4	2.09E-04	T-cell lymphoma invasion and metastasis 1
tnip1	1.6	5.77E-03	1.3	6.00E-04	1.4	2.39E-04	TNFAIP3 interacting protein 1
traf1	1.2	1.08E-02	1.6	2.77E-06	1.4	8.16E-04	Tnf receptor-associated factor 1
cd40	1.6	8.85E-01	1.5	2.35E-01	1.3	3.05E-01	CD40 antigen
ebi3	1.0	3.37E-01	1.2	2.42E-03	1.3	4.24E-03	Epstein-Barr virus induced gene 3
gpr84	1.1	8.53E-02	1.5	1.27E-04	1.3	2.47E-02	G protein-coupled receptor 84
mmp13	5.0	1.03E-06	1.9	6.39E-02	1.3	1.64E-01	matrix metallopeptidase 13
osm	1.0	9.90E-01	1.1	4.74E-02	1.3	4.28E-03	oncostatin M
pilrb1	1.0	2.54E-01	1.3	2.22E-03	1.3	5.05E-03	paired immunoglobin-like type 2 receptor beta 1
ppnr	1.1	1.22E-01	1.2	3.50E-02	1.3	3.97E-03	per-pentamer repeat gene
spic	1.1	4.88E-01	1.3	7.35E-03	1.3	9.31E-02	Spi-C transcription factor
tcf7l2	1.0	2.67E-01	1.1	1.49E-02	1.3	1.29E-03	transcription factor 7-like 2, T-cell specific, HMG-box
tnfaip2	1.4	8.97E-03	1.0	9.09E-01	1.3	4.32E-02	tumor necrosis factor, alpha-induced protein 2
2610027c15rik	1.2	1.76E-02	1.1	2.12E-01	1.2	2.07E-02	RIKEN cDNA 2610027C15 gene
adora2b	1.2	4.70E-03	1.3	7.90E-04	1.2	5.89E-02	adenosine A2b receptor
arg2	1.0	2.15E-01	1.2	4.86E-02	1.2	6.08E-02	arginase type II
cd33	1.0	7.87E-01	1.4	7.39E-06	1.2	1.95E-02	CD33 antigen
flrt3	1.0	3.51E-01	1.4	8.03E-03	1.2	4.11E-02	fibronectin leucine rich transmembrane protein 3
loc100048721	1.1	1.70E-01	1.3	5.34E-03	1.2	4.41E-02	similar to fibronectin leucine rich transmembrane protein 3
slco3a1	1.2	4.29E-01	1.2	2.00E-01	1.2	8.91E-01	solute carrier organic anion transporter family, member 3a1
zdhhc14	1.1	1.28E-01	1.1	8.25E-02	1.2	3.52E-02	zinc finger, DHHC domain containing 14
clec4e	1.3	3.23E-03	1.4	6.68E-04	1.1	1.05E-01	C-type lectin domain family 4, member e
micall2	1.1	2.59E-01	1.3	2.05E-03	1.1	6.47E-02	MICAL-like 2
plagl2	1.1	1.36E-01	1.2	5.22E-03	1.1	1.67E-01	pleiomorphic adenoma gene-like 2
slc31a2	1.0	6.87E-01	1.3	7.77E-04	1.1	7.59E-02	Solute Carrier Family 31 (Copper Transporter), Member 21
tug1	1.0	8.76E-01	1.1	8.88E-01	1.1	7.83E-01	taurine upregulated gene 1
csf1	1.4	8.84E-03	1.5	7.07E-04	1.0	5.99E-01	colony stimulating factor 1
tmem132e	1.2	1.94E-02	1.2	4.67E-03	1.0	8.93E-01	transmembrane protein 132E
pld4	1.1	4.52E-02	1.3	6.23E-04	-2.0	5.35E-08	phospholipase D family, member 4
lpl	1.5	3.20E-03	-2.5	2.93E-06	-5.2	1.82E-12	lipoprotein lipase

Gene expression (fold change; FC), with associated p-values, relative to control tissues in the *ex vivo* native mouse liver model over 48 hours after the addition of SEA determined using t-tests with multiple testing correction p≤0.05 (Benjamin-Hochberg).

In addition to the genes identified by t-tests, novel genes were also identified using either a 2 fold cut off or due to their known action in the host immune system or in tissue remodeling (Table 2). A variety of chemokines were evident including CCL2 & 7, CXCL1 & 16, and cytokines IL 6, 10 & 11, while genes associated with extracellular matrix degradation included MMP 3, 9, 10 & 13, and TIMP 1. Generally, all genes were up-regulated (Table 2) with differential expression increasing with time over the 48hrs in culture with the addition of SEA. Genes related directly to collagen deposition were generally unchanged in the presence of SEA with moderate (<1.4 fold) up-regulation observed at 48hrs in culture (S2 Table). Some genes were down-regulated during the time course such as AQP1 (aquaporin 1, -3.1 fold at 24hrs); LPL (lipoprotein lipase, -5.2 fold at 48hrs); EAR2 (eosinophil-associated ribonuclease A family member 2, -3.5 fold at 48hrs); and VSIG4 (V-set and immunoglobulin domain containing 4, -4 fold at 48hrs) (See S2 Table).

**Table 2:** Novel differentially expressed genes in response to *S*. *japonicum* soluble egg antigen (SEA).

	FC SEA / control (hours)						
Symbol	4	p-Value	24	p-Value	48	p-Value	Definition
angpt2	-3.1	1.20E-05	1	9.96E-01	1.1	1.00E+00	angiopoietin 2
aqp1	1	4.78E-01	-3	1.40E-04	-2.3	2.80E-03	aquaporin 1
ccl2	1.8	9.19E-03	1.7	1.47E-02	1.3	1.00E+00	chemokine (C-C motif) ligand 2
ccl7	1.1	6.38E-01	1.8	3.38E-02	1.7	1.00E+00	chemokine (C-C motif) ligand 7
ccrl2	1.4	1.44E-02	1.5	1.14E-02	1.2	1.00E+00	chemokine (C-C motif) receptor-like 2
cd14	1.3	1.07E-01	1.2	1.70E-01	1.9	6.04E-04	CD14 antigen
col1a1	-1.1	2.65E-01	-1.1	2.61E-01	1.4	6.64E-03	procollagen, type I, alpha 1
col5a1	1.1	3.80E-01	1	8.40E-01	1.7	3.41E-01	procollagen, type V, alpha 1
cxcl1	1.1	2.60E-01	1	7.51E-01	2.2	2.30E-05	chemokine (C-X-C motif) ligand 1
cxcl16	1.6	2.10E-03	1.5	3.18E-04	1.3	9.30E-01	chemokine (C-X-C motif) ligand 16
fstl1	1	8.81E-01	-1.1	2.61E-01	1.7	1.26E-01	follistatin-like 1
ifit3	-1.2	4.36E-01	2.3	3.09E-02	1	1.00E+00	interferon-induced protein with tetratricopeptide repeats 3.
iigp2	-1.5	1.91E-03	1.8	1.74E-06	1.1	1.00E+00	interferon inducible GTPase 2
il10	1.9	6.79E-08	1.2	4.53E-03	1.2	1.00E+00	interleukin 10
il11	-1.3	8.55E-02	-1.2	3.57E-01	2.4	1.06E-04	interleukin 11
il4i1	1.8	1.09E-06	1.2	3.72E-03	1.3	9.75E-01	interleukin 4 induced 1
il6	-1.1	3.87E-01	1	9.50E-01	2.3	4.17E-03	interleukin 6
irak3	1.3	8.93E-03	1.8	9.76E-05	2.6	3.34E-05	interleukin-1 receptor-associated kinase 3
loc100044702	-1.2	2.05E-01	1.1	5.54E-01	2.7	4.19E-03	similar to LPS-induced CXC chemokine.
ly6a	-1.4	3.47E-03	2.1	7.11E-04	1	1.00E+00	lymphocyte antigen 6 complex, locus A
mmp10	1	7.21E-01	1.3	1.06E-01	1.8	1.00E+00	matrix metallopeptidase 10
mmp3	1	9.65E-01	-1.1	7.73E-01	1.7	7.94E-02	matrix metallopeptidase 3
mmp9	1	6.94E-01	1.4	1.14E-01	2.1	3.44E-03	matrix metallopeptidase 9
pglyrp1	-1.1	3.78E-01	1.2	1.24E-02	1.7	3.12E-03	Peptidoglycan recognition protein
serpinb2	1.1	1.11E-01	1.1	5.22E-01	2.5	4.89E-05	serine (or cysteine) peptidase inhibitor, clade B, member 2.
sod2	1	9.87E-01	1.2	3.69E-02	1.8	9.06E-03	Superoxide dismutase 2, mitochondrial
timp1	-1.1	7.66E-01	-1.1	5.10E-01	2.1	2.76E-03	tissue inhibitor of metalloproteinase 1
tnc	1	6.29E-01	-1.2	1.28E-01	2.3	9.88E-02	tenascin C
tnf	1.9	2.27E-04	1.3	3.90E-03	1.2	1.00E+00	tumor necrosis facto
tlr4	-1.1	1.94E-01	1	9.19E-01	1	1.00E+00	toll-like receptor 4

Gene expression fold changes (FC) with associated p-value, relative to control tissue in *ex vivo* native mouse liver model over 48 hours after the addition of SEA. Genes with known roles in immune responses or tissue remodeling are shown.

### Pathway analysis

To better appreciate the broader biological significance of the 48 genes identified as differentially expressed in the presence of SEA, Ingenuity Pathway Analysis (IPA) was undertaken. Presented in Table 3, the top 20 canonical pathways based on p-value, included those related to granulocyte, macrophage and hepatic stellate cell activity. Immunological activities were associated with cytokine production and IL-10 and IL-17, and broad terms including acute phase and communication between innate and adaptive responses. Gene pathways of liver specific functions modulated by SEA including cholestasis and liver X receptor (LXR) signalling were also identified. A complete summary of the entire IPA analysis, including all canonical pathways (126) identified, upstream regulators, networks, toxicity lists and specific molecules, is presented in S3 Table.

**Table 3:** Top 20 pathways identified by Ingenuity Pathway Analysis.

Ingenuity Canonical Pathways	P-value	Genes
Granulocyte Adhesion and Diapedesis	4.37E-09	CXCL3, IL1A, CCL4, FPR2, CCL3L1/CCL3L3, MMP13, IL1B, FPR1
Atherosclerosis Signaling	9.55E-09	IL1A, CD40, CSF1, LPL, MMP13, IL1B, S100A8
Role of Macrophages, Fibroblasts and Endothelial Cells in Rheumatoid Arthritis	2.04E-08	TLR2, IL1A, CSF1, MMP13, OSM, IL1B, IRAK3, TCF7L2, TRAF1
Communication between Innate and Adaptive Immune Cells	5.13E-08	TLR2, IL1A, CCL4, CD40, CCL3L1/CCL3L3, IL1B
Hepatic Fibrosis / Hepatic Stellate Cell Activation	6.46E-07	CXCL3, IL1A, CD40, CSF1, MMP13, IL1B
Altered T Cell and B Cell Signaling in Rheumatoid Arthritis	1.35E-06	TLR2, IL1A, CD40, CSF1, IL1B
NF-Î°B Signaling	2.29E-06	TLR2, IL1A, TNIP1, CD40, IL1B, IRAK3
Agranulocyte Adhesion and Diapedesis	3.39E-06	CXCL3, IL1A, CCL4, CCL3L1/CCL3L3, MMP13, IL1B
LXR/RXR Activation	7.41E-06	IL1A, LPL, IL1B, S100A8, ARG2
Differential Regulation of Cytokine Production in Intestinal Epithelial Cells by IL-17A and IL-17F	1.82E-05	IL1A, CCL4, IL1B
TREM1 Signaling	2.40E-05	TLR2, CXCL3, CD40, IL1B
Role of Osteoblasts, Osteoclasts and Chondrocytes in Rheumatoid Arthritis	1.29E-04	IL1A, CSF1, MMP13, IL1B, TCF7L2
Role of Hypercytokinemia/hyperchemokinemia in the Pathogenesis of Influenza	1.32E-04	IL1A, CCL4, IL1B
Role of IL-17F in Allergic Inflammatory Airway Diseases	1.41E-04	CCL4, MMP13, IL1B
Hepatic Cholestasis	2.63E-04	IL1A, SLCO3A1, IL1B, IRAK3
Toll-like Receptor Signaling	2.69E-04	TLR2, IRAK3, TRAF1
Role of IL-17A in Psoriasis	3.80E-04	CXCL3, S100A8
IL-10 Signaling	4.79E-04	IL1A, IL1B, ARG2
Acute Phase Response Signaling	5.50E-04	IL1A, Saa3, OSM, IL1B
Dendritic Cell Maturation	6.76E-04	TLR2, IL1A, CD40, IL1B

From the 48 differentially expressed genes in response to *S*. *japonicum* soluble egg antigen in the *ex vivo* native mouse liver model over 48 hours.

### Real-time PCR validation of microarray findings

**Fig 3:**
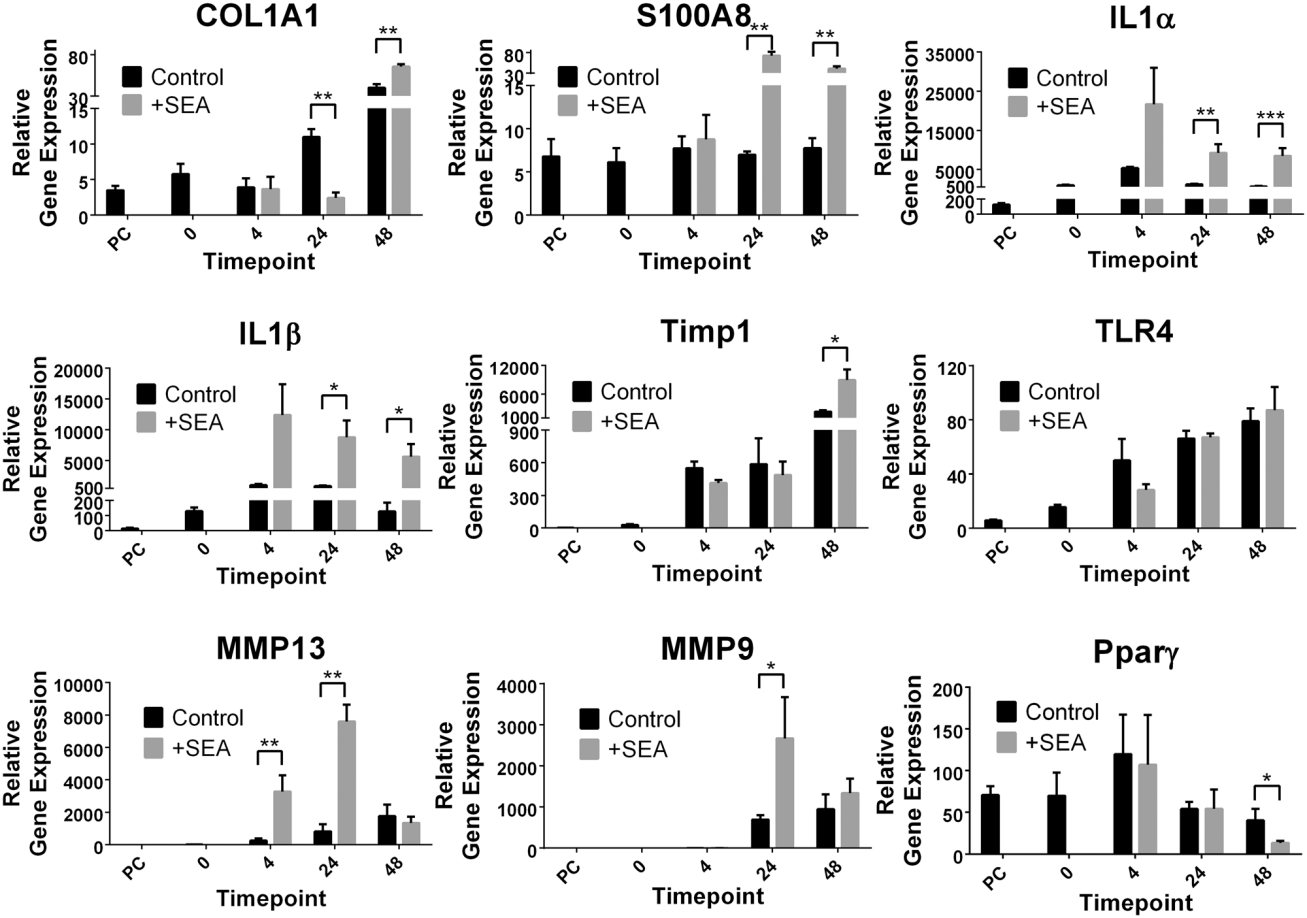
Real time validation of a subset of the 13,746 genes. Genes were identified both as differentially expressed and unchanged due to the presence of soluble egg antigen (SEA) from *Schistosoma japonicum*. Relative gene expression was normalised to the house keeping gene HPRT. Time points refer to pre-cut intact tissue (PC) and tissue slices in culture before (0) and after (4, 24 and 48 hours) the addition of soluble egg antigen (SEA). P values = *≤0.05, **≤0.01, ****≤0.0001.

To validate the microarray findings, a subset of nine genes was analysed using qPCR. The relative fold change of gene expression determined by microarray analysis and qPCR were overall similar for the majority of data points for all nine genes and three time points (Fig 3, Tables 1 and 2). The microarray and qPCR data for the nine genes showed a significant correlation (-α = 0.05) between the two methods (Spearman’s Rho = 0.9435, P value = <0.0001, *n* = 27), indicating considerable support for the results obtained by the microarray analysis. The major difference noted between the qPCR and microarray findings was the down-regulation of COL1a1 (collagen type I -α 1) (-4.5 fold) in the presence of SEA at 24hrs; however by 48hrs this decrease had changed to a moderate (1.6 fold) but significant (p value ≤ 0.01) increase in gene expression (Fig 3).

### Detection of chemokines and cytokines in culture supernatant

**Fig 4:**
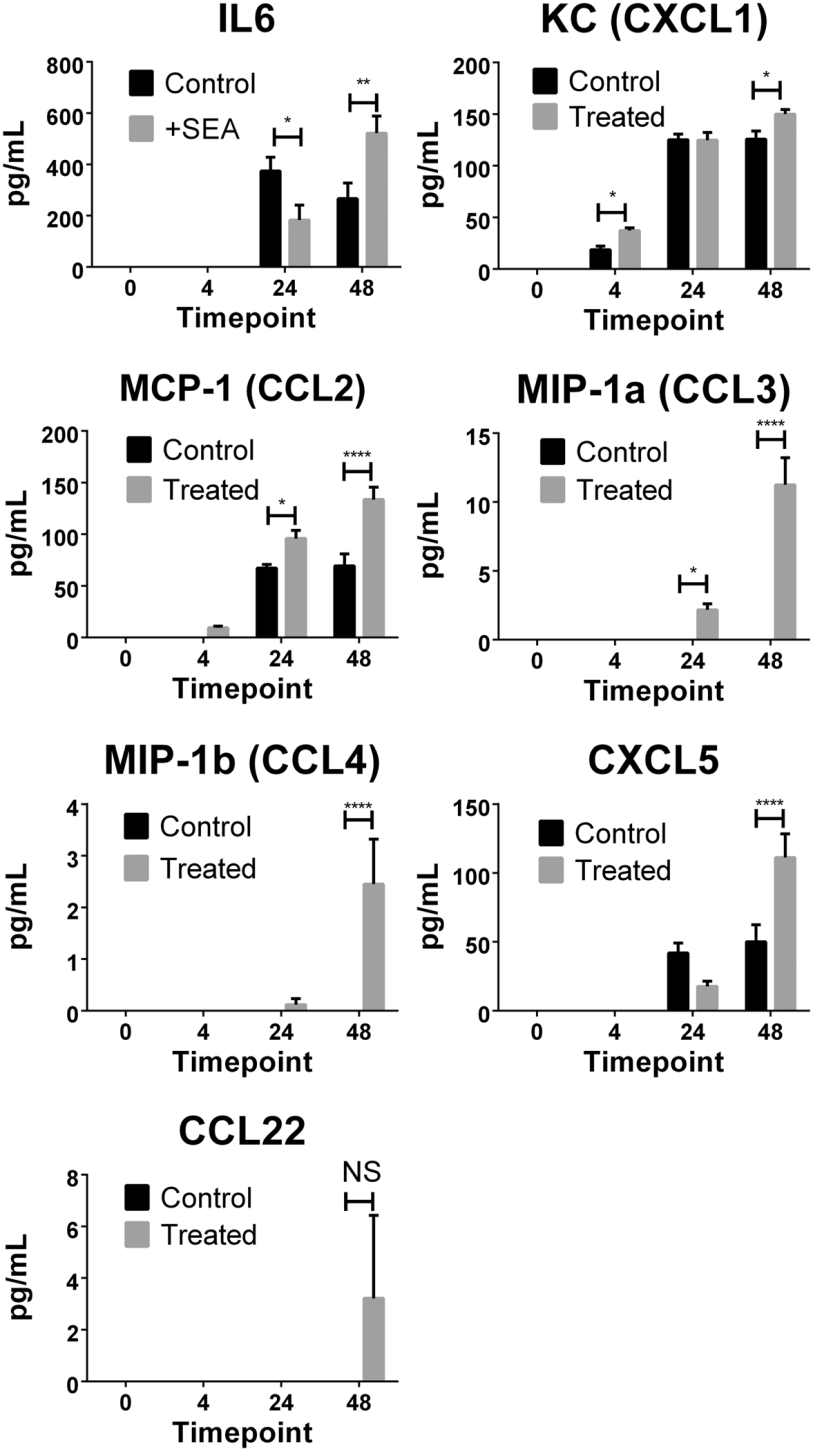
Cytokine and chemokine levels were determined in liver section culture supernatants. Using Legendplex multi-analyte flow assay kits, IL-6, CXCL1, CCL2, CCL3, CCL4, CXCL5 and CCL22 were detected. Levels of significance were calculated using two-way ANOVA with Sidaks multiple comparison test. P values = *≤0.05, **≤0.01, ****≤0.0001, NS = not significant >0.05.

To further validate the microarray findings, the culture supernatants were assessed for a subset of 13 chemokines and 13 cytokines using multi-analyte flow assays. Seven proteins were detected in the culture supernatant and all were shown to be differentially expressed between SEA-treated and untreated samples (Fig 4). Of the thirteen cytokines measured, only IL-6 was detected in the culture supernatant at 24 and 48 hours post treatment. Significantly more IL-6 was measured in SEA-treated groups at 48 hours when compared with untreated groups, which correlated with the transcriptional data obtained. Chemokines CXCL1, CCL2 and CXCL5 were detected in culture supernatants from both SEA-treated and untreated liver samples, with elevated expression of these proinflammatory molecules after SEA treatment, as observed in the differential gene expression measured by microarray (Table 2). Detectable levels of CCL3, CCL4 and CCL22 were measured only in the supernatant of SEA-treated liver sections. These finding mirrored observed increases in gene expression for these molecules in the microarray analysis (Table 1). The close correlation of protein measurement in the supernatants with the gene expression data from the microarray supports the validity of our findings.

## Discussion

The precision cut liver slice (PCLS) mouse model [17] is a novel approach to study schistosomiasis that uniquely allows the investigation of the early local responses of a mixture of residential hepatic cells (hepatic stellate cells—HSCs, hepatocytes and KCs etc) to soluble molecules released by dying schistosome eggs. Using this system we have shown how early molecular events contribute to the eventual granulomatous response that takes place in the liver of the *S*. *japonicum* infected definitive host. We consider our findings represent the early interaction of dying schistosome eggs, and the release of all of their soluble protein (SEA) into the host liver microenvironment. There is, however, a much longer process that leads to granuloma formation [1,3], involving non-residential cell recruitment and cytokine signalling, and eventual chronic disease. As such, *in vivo* studies are necessary to follow disease progression over the time period that follows.

These *ex vivo* observations, build on our previously published *in vivo* studies using transcriptional profiling of naturally infected whole livers [2,28]. Furthermore, these *in vivo* results identified the contribution of hepatic immune cell types in the resulting granuloma formation in host liver, and shed light on the initial molecular interaction between parasite antigen and residential hepatic cells. The MDA concentration in the media of PCLS did not increase due to the presence of SEA, but did increase during the 48hrs in culture. However the increase seen is comparable to previous studies where other related liver enzymes were measured and controls increased between 1.5 and 3 fold over a similar period in culture [29]. One potential improvement to our *ex vivo* model that could enhance the fidelity of the tissues, would be the use of hyper-oxygenation to the culture conditions using specialised equipment [30]. The use of hyperbaric culture conditions could also be considered as these have protective properties in reducing mitochondrial damage [31]. The impact of oxidative stress on hepatic pathology and immunology may be a factor and while the *ex vivo* conditions we utilised were sub-optimal, they have provided an avenue to investigate hepatic schistosomiasis in a complex tissue model that is a considerable improvement on traditional *in vivo* studies, being able to synchronise the delivery of parasite antigen to liver tissue.

PCLS have been successfully employed in studies of liver metabolism and toxicity [18]. Fibrosis has also been investigated using PCLS, predominantly using chemical or surgical methods [18]. The application of an *ex vivo* model to investigate tissue remodeling in the liver in response to a major human pathogen, is a novel approach.

While schistosome eggs that lodge in the liver of hosts produce extensive fibrotic granuloma, no fibrosis is evident local to the egg [1–3,22]. When exposed to schistosome eggs, the profibrogenic phenotype of a human HSC line (LX-2 cells) is blocked, i.e., collagen production is switched off and a state of quiescence is induced [12,13]. This demonstrates that schistosome eggs produce mediators with potent and novel anti-fibrotic activity on the major collagen producing cells of the liver. Our observation by qPCR of a decrease in Col1A1 in the presence of SEA supports this observation.

One of the highest up-regulated genes identified was serum amyloid A 3 (SAA3) which has been linked previously to liver disease [32]. The expression of SAA3 has been associated with the metastasis of cancer cells and a corresponding influx of immune cells [33]. Proteins of the SAA family, including SAA3, have been shown to enhance the expression of a number of proinflammatory proteins such as S100a8, and various matrix metalloproteinases (MMPs) [33]. Furthermore, other studies have linked SAA to formyl peptide receptor [34] and this also drives the expression of MMPs, while the SAA inflammation pathway mediates IL-1-β secretion [35]. Proinflammatory S100a8, IL-1-β and FPR2, and the tissue remodeling MMP3, 9, 13 were all up-regulated in the *ex vivo* model we used, supporting these potential molecular mechanisms.

As shown in mouse models of liver damage, the TLR2/S100A9/CXCL-2 signaling network drives neutrophil recruitment [36]. CXCL2 has been shown in low concentrations to provide protection against cell death, while at higher levels it induces apoptosis in hepatocytes [37]. The elevation of TLR2, CXCL-2 and S100A8, with the latter originally demonstrated using the knockout of S100A9 which leads to S100A8 protein instability, suggests that the earliest exposure of parasite antigen leads to the recruitment of neutrophils to the liver. This mirrors the well reported neutrophilic nature of hepatic granulomas that are distinctive in infections of *S*. *japonicum* while absent from those of *S*. *mansoni* [2,22,38]. However, in our *ex vivo* system the exclusion of circulatory cells allows us to focus clearly on residential cells, including those associated with the innate immune system. In addition to the previously mentioned role in MMP expression, the elevation of FPR2 may also increase neutrophil recruitment since the release of a related formyl peptide receptor (FPR1) from necrotic tissues appears distinct from the function of CXCL family of chemokines ([39], and reviewed in [40]).

The observed up-regulation of various MMPs in our *ex vivo* system may be in response to the corresponding up-regulation of TNC (Tenascin-C). TNC is an extracellular matrix protein associated with cellular adhesion having a direct interaction with fibronectin, and results in the up-regulation of MMPs [41]. However, the temporal expression of TNC and MMPs did not clearly demonstrate their relationship since TNC was only elevated at 48 hrs while MMP 9&10 were both increased at 24hrs (see Table 2). Additional study and validation of the transcriptional and protein levels of these genes in response to schistosome antigens will further our understanding of their importance in tissue remodeling during hepatic schistosomiasis. In our study, other indicators of tissue hepatic remodeling is seen in the decrease in AQP1 expression, -3 fold after 24hrs and -2.3 fold after 48hrs. Aquaporin 1 is expressed in proliferating bile ducts [42] however reduced levels are associated with intrahepatic cholestasis, and a decrease in bile flow. A feature of hepatic schistosomiasis is the decrease in bile flow [43] and the decrease in AQP1 expression we observed *ex vivo* may mirror the *in vivo* clinical setting.

Interleukin 1 (IL-1-β and IL-1-α) had amongst the largest increase in gene expression over the 48hrs after SEA exposure of liver slices in culture. Proinflammatory cytokines such as IL-1 are primarily expressed in the liver by monocytes, KCs and peripheral neutrophils. Increases in cellular stress in hepatocytes can induce necrosis, which leads to the expression of IL-1-α [44] and the activation of KCs which themselves produce IL-6. Thus, the elevated IL-1-β and IL-1-α observed in our system were particularly striking due to the exclusion of recruited leukocytes in our *ex vivo* model.

The observed correlation of chemokines and cytokines measured in the culture supernatants with the gene expression data collected in the microarray supports our gene expression data. The observation of an increase in only one (IL-6) of the 13 cytokines investigated is not surprising in the context of our infection model. As the liver tissue was harvested from uninfected animals and then isolated in an *ex vivo* culture system, the likelihood of detecting T cell derived cytokines was minimal. Any T cells would first need to be activated by antigen specifically and migrate into the tissue before producing measurable cytokine. The detection of IL-6 demonstrates a robust response to immune assault; IL-6 is produced by various cell types, such as endothelial cells and fibroblasts, and can be secreted by macrophages in response to PAMPs (pathogen associated molecular patterns). The early inflammatory cytokines IL-1-α and IL-1-β are known to induce up-regulation of IL-6 and they were shown to have strongly increased expression from as early as 4hrs post SEA exposure (Table 1 and Fig 3).

Inflammatory macrophages and also TLR4 activated HSCs release the chemokines CCl3 (2.8|2.3|2.4; fold changes at 4hrs, 24hrs, 48hrs), Ccl4 (2.0|1.5|1.6), which acts on hepatic stellate cell proliferation [45], and recruitment of KCs [46], leukocytes and monocytes [47] to regions of cellular damage. We were able to directly measure CCL3 and CCL4 among other proinflammatory cytokines in culture supernatants. The increased levels of proinflammatory chemokines reflect a strong response to SEA, with the induced chemokines having important roles in the trafficking and recruitment of immune cells. The strong up-regulation of these proinflammatory chemokines, early after SEA exposure, demonstrates that SEA induces clear danger signals in resident liver cells, which in turn drive the recruitment of the specialised immune cells required to form the granulomatous lesions that will eventually encase schistosome eggs in an infection.

Activated macrophages produce TNF-α (1.9 fold at 4hrs) and IL-1 (consistently up-regulated across time course) that in turn activate fibroblasts and induce overproduction of ECM proteins (1.2 fold at 4hrs, 1.4 fold at 24hrs, and 1.7 fold at 48hrs). TNF-α-triggered (1.9 fold at 4hrs) signal transduction leading to the expression of fibrogenic responses.

Activation of KCs in our *ex vivo* model was reflected in the up-regulation of TNF (1.9|1.3|1.2), IL6, IL1 and CD40 (1.6|1.5|1.3). IRAK (interleukin-1 receptor-associated kinase) is a family of genes encoding proteins which are important components of immune signal transduction pathways [48]. Associated with Toll/IL-R signalling, IRAKs are expressed primarily in monocytes and macrophages and act as negative regulators. The CD14-TLR2-IRAK4 complex is associated with KC when LPS triggers TLR4 signaling which resulted in the production of TNF (1.9 fold at 4 h), IL-1b (4.1|5.2|10.7), IL-6 (-1.1|1|2.3), IL-12 (not detected), IL-18 (unchanged), and IL-10 (1.9|1.2|1.2) [49]. In our experiments, CD14 (1.9 fold) and TLR2 (1.8 fold) were both up-regulated after 48hrs but IRAK4 was unchanged. KCs also express TLR2 (1.4|1.4|1.8), TLR3, and TLR9 [50], with the latter two genes not presenting any detectable expression in our microarray analysis of liver slices exposed to SEA. While IRAK4 (and IRAK1&2) was unchanged in our experiments, the related gene IRAK3 was up-regulated 2.6 fold at 48 h. IRAK3 (IRAK-M), unlike IRAK1,2 and 4 has limited capacity to act in pro-inflammatory complexes, but instead acts as a negative regulator acting on IRAK1 and 2 disrupting their complex formation, and effectively reducing downstream inflammatory mechanisms [48]. Again our *ex vivo* model reflected the gene expression of predominantly residential cells of the liver, with only the presence of basal amounts of circulating cells present in the processing of precision cut liver slices.

There were indications of the presentation of antigen in the exposure of liver slices to SEA. This was reflected in the up-regulation of the following genes: CCL4, CD40, CLEC4E, CSF1, CXCL3, IL1B, OSM and TLR2, all of which are associated with the recruitment of antigen presenting cells (as identified by IPA; see S3 Table). The release of CCL3 (2.9|2.4|2.5) has been reported in KCs to recruit dendritic cells [51], which suggests a potential mechanism that SEA induces in the residential cells in our *ex vivo* model.

The early increase in MMP13 gene expression at 4hrs which decreased over time (5|1.9|1.3), coupled with the opposite expression of TIMP1 which was unchanged at 4 and 24hrs and then increased at 24hrs (-1.1|-1.1|2.1) may explain the decrease in collagen deposition which was observed in the direct core of schistosome-induced granuloma. The initial exposure to SEA led to reduced TIMP1 and increased MMP13, which would limit fibrosis deposition. The increase in TIMP1 observed at the latest time point mirrors the clinical situation where elevated levels are associated with hepatic fibrosis due to schistosomiasis japonica in supernatents of patient PBMCs (peripheral blood mononuclear cells) stimulated with schistosome egg antigen [52]. TIMP1 was also elevated in experiments using trophoblast cells co-cultured with plasma from pregnant women with schistosomiasis history or direct stimulation with SEA [53]. These investigations and a subsequent study of neonatal cord blood [54], demonstrate that schistosome egg antigens induce a pro-inflammatory response during pregnancy and in newborns of schistosome infected mothers. No correlation of MMPs (MMP1) with the development of hepatic fibrosis was evident in the PBMC study [52], but the cord blood analysis did show significant increases in the production of MMP2 during fibrosis [54]. This work and our observations in naïve liver tissue exposed to SEA demonstrate the importance of inflammatory responses and tissue remodeling markers in schistosomiasis, both at the primary site of pathology and, systemically, especially during pregnancy.

We propose that the *ex vivo* model we have employed represents well the interaction between liver cells and the release of SEA that occurs when schistosome eggs are deposited *in vivo* and subsequently die. Our transcription data obtained from *ex vivo* maintained tissue supports this correlation with the *in vivo* situation when we consider our previously published findings [22]. In the previous study we used laser microscopy microdissection (LMM) to isolate specific regions within and proximal to the granuloma formed in the livers of infected *S*. *japonicum* mice. However it must be noted that *in vivo* granuloma formation following the deposition of parasite eggs and the release of SEA, would be accompanied by the influx of peripheral cells, a feature that is not present in the *ex vivo* model. However despite these clear differences between models, many genes were up-regulated in both studies including those associated with acute inflammation, such as SAA3, Type-1 genes, such as IL1-α, CCL4, CXCL2 and Type-17 genes, such as IL6, IL1-β, CCL3.

While this study has utilised naïve wild type mouse liver tissue, the future value of this model could lie in the use of transgenic or cell depleted host tissue, to better define the specific components of the residential cells that lead to inflammation, antigen presentation and fibrosis in response to SEA. Further studies could focus also on the effect on the *ex vivo* model using earlier released schistosome proteins in egg ES (excretory/secretory) products. These can be readily collected from the media of isolated and then cultured live parasite eggs. This subset of schistosome egg proteins would represent the very first stages of egg deposition in the liver of the host, demonstrating the interaction the hepatic environment has with live schistosome eggs. Our findings provide a unique snapshot of the earliest phases of hepatic schistosomiasis, where resident hepatic cells are first exposed to parasite egg antigen (SEA). Further, the findings from this *ex vivo* approach have far reaching potential consequences in other hepatic pathologies defined by fibrosis including alcoholic cirrhosis and viral infections.

## Supporting Information

S1 Table Primer list used for real time PCR analysis of gene subset. (XLSX)

S2 Table All 13,746 genes identified after filtering for flags and detection score. Column A- Gene Symbol. Columns B/C- Gene expression fold Change (FC) and associated p-value, at 4hrs for liver tissue exposed to soluble egg antigen (SEA) relative to control liver tissue at the same time point. Columns D/E- FC and associated p-value, at 24hrs for liver tissue exposed to SEA relative to control liver tissue at the same time point. Columns F/G- FC and associated p-value, at 48hrs for liver tissue exposed to SEA relative to control liver tissue at the same time point. Column H- Gene definition. Column I- Illumina microarray identifier. Column J- NCBI Reference Sequence (RefSeq) identifier. Column K- Synonyms or alternative gene names. Column L Genes identified from 1 Way ANOVA based on Time.
(XLSX)

S3 Table Ingenuity Pathway Analysis (IPA) of the 48 genes identified as differentially expressed in the presence of SEA was undertaken. The complete summary of the entire analysis, including all canonical pathways (126) identified, upstream regulators, networks, toxicity lists and specific molecules are included.
(XLSX)

## References

[1] Burke ML, Jones MK, Gobert GN, Li YS, Ellis MK, McManus DP Immunopathogenesis of human schistosomiasis. Parasite Immunol. 2009;31: 163–176. 10.1111/j.1365-3024.2009.01098.x 19292768

[2] Burke ML, McManus DP, Ramm GA, Duke M, Li Y, Jones MK, et al Temporal expression of chemokines dictates the hepatic inflammatory infiltrate in a murine model of schistosomiasis. PLoS Negl Trop Dis. 2010;4: e598 10.1371/journal.pntd.0000598 20161726 PMC2817718

[3] Chuah C, Jones MK, Burke ML, McManus DP, Gobert GN Cellular and chemokine-mediated regulation in schistosome-induced hepatic pathology. Trends Parasitol. 2014;30: 141–150. 10.1016/j.pt.2013.12.009 24433721

[4] Pearce EJ, MacDonald AS The immunobiology of schistosomiasis. Nat Rev Immunol. 2002;2: 499–511. 12094224 10.1038/nri843

[5] Wilson MS, Mentink-Kane MM, Pesce JT, Ramalingam TR, Thompson R, Wynn TA Immunopathology of schistosomiasis. Immunol Cell Biol. 2007;85: 148–154. 17160074 10.1038/sj.icb.7100014 PMC3437548

[6] Smyth JD, Halton DW (1983) The physiology of trematodes: Cambridge University Press 110 p.

[7] Gryseels B, Polman K, Clerinx J, Kestens L Human schistosomiasis. Lancet. 2006;368: 1106–1118. 16997665 10.1016/S0140-6736(06)69440-3

[8] Pesce JT, Ramalingam TR, Wilson MS, Mentink-Kane MM, Thompson RW, Cheever AW, et al Retnla (relmalpha/fizz1) suppresses helminth-induced Th2-type immunity. PLoS Pathog. 2009;5: e1000393 10.1371/journal.ppat.1000393 19381262 PMC2663845

[9] Thohan S, Rosen GM Liver Slice Technology as an *In Vitro* Model for Metabolic and Toxicity Studies. Methods in Molecular Biology. 2002;196: 291–303. 12152207 10.1385/1-59259-274-0:291

[10] Bartley PB, Ramm GA, Jones MK, Ruddell RG, Li Y, McManus DP A contributory role for activated hepatic stellate cells in the dynamics of *Schistosoma japonicum* egg-induced fibrosis. Int J Parasitol. 2006;36: 993–1001. 16806222 10.1016/j.ijpara.2006.04.015

[11] Chang D, Ramalho LN, Ramalho FS, Martinelli AL, Zucoloto S Hepatic stellate cells in human schistosomiasis mansoni: a comparative immunohistochemical study with liver cirrhosis. Acta Trop. 2006;97: 318–323. 16473318 10.1016/j.actatropica.2005.12.006

[12] Anthony B, Mathieson W, de Castro-Borges W, Allen J *Schistosoma mansoni*: egg-induced downregulation of hepatic stellate cell activation and fibrogenesis. Exp Parasitol. 2010;124: 409–420. 10.1016/j.exppara.2009.12.009 20045695

[13] Anthony BJ, James KR, Gobert GN, Ramm GA, McManus DP *Schistosoma* Eggs Induce a Proinflammatory, Anti-Fibrogenic Phenotype in Hepatic Stellate Cells. PLoS ONE. 2013;8: e68479 23840855 10.1371/journal.pone.0068479 PMC3686876

[14] Liu P, Wang M, Lu XD, Zhang SJ, Tang WX *Schistosoma japonicum* egg antigen up-regulates fibrogenesis and inhibits proliferation in primary hepatic stellate cells in a concentration-dependent manner. World J Gastroenterol. 2013;19: 1230–1238. 10.3748/wjg.v19.i8.1230 23482848 PMC3587479

[15] Duan Y, Gu X, Zhu D, Sun W, Chen J, Feng J, et al *Schistosoma japonicum* soluble egg antigens induce apoptosis and inhibit activation of hepatic stellate cells: a possible molecular mechanism. Int J Parasitol. 2014;10 doi: 1016/j.ijpara.2013.11.003 10.1016/j.ijpara.2013.11.003 24487000

[16] Boess F, Kamber M, Romer S, Gasser R, Muller D, Albertini S, et al Gene expression in two hepatic cell lines, cultured primary hepatocytes, and liver slices compared to the *in vivo* liver gene expression in rats: possible implications for toxicogenomics use of *in vitro* systems. Toxicol Sci. 2003;73: 386–402. 12657743 10.1093/toxsci/kfg064

[17] Duryee MJ, Willis MS, Schaffert CS, Reidelberger RD, Dusad A, Anderson DR, et al Precision-cut liver slices from diet-induced obese rats exposed to ethanol are susceptible to oxidative stress and increased fatty acid synthesis. Am J Physiol Gastrointest Liver Physiol. 2014;306: G208–217. 10.1152/ajpgi.00124.2013 24284960 PMC3920111

[18] Olinga P, Schuppan D Precision-cut liver slices: a tool to model the liver ex vivo. J Hepatol. 2013;58: 1252–1253. 10.1016/j.jhep.2013.01.009 23336979

[19] Perry CR, Burke ML, Stenzel DJ, McManus DP, Ramm GA, Gobert GN Differential expression of chemokine and matrix re-modelling genes is associated with contrasting schistosome-induced hepatopathology in murine models. PLoS Negl Trop Dis. 2011;5: e1178 10.1371/journal.pntd.0001178 21666794 PMC3110159

[20] Guo Y, Wu XQ, Zhang C, Liao ZX, Wu Y, Xia ZY, et al Effect of indole-3-carbinol on ethanol-induced liver injury and acetaldehyde-stimulated hepatic stellate cells activation using precision-cut rat liver slices. Clin Exp Pharmacol Physiol. 2010;37: 1107–1113. 10.1111/j.1440-1681.2010.05450.x 20880187

[21] Jimenez-Marin A, Collado-Romero M, Ramirez-Boo M, Arce C, Garrido JJ Biological pathway analysis by ArrayUnlock and Ingenuity Pathway Analysis. BMC Proc. 2009;3 Suppl 4: S6 10.1186/1753-6561-3-S4-S6 19615119 PMC2712749

[22] Chuah C, Jones MK, Burke ML, Owen HC, Anthony BJ, McManus DP, et al Spatial and temporal transcriptomics of *Schistosoma japonicum*-induced hepatic granuloma formation reveals novel roles for neutrophils. J Leukoc Biol. 2013;94: 353–365. 10.1189/jlb.1212653 23709687

[23] Ganguly K, Rejmak E, Mikosz M, Nikolaev E, Knapska E, Kaczmarek L Matrix metalloproteinase (MMP) 9 transcription in mouse brain induced by fear learning. J Biol Chem. 2013;288: 20978–20991. 10.1074/jbc.M113.457903 23720741 PMC3774367

[24] Fukuda H, Mochizuki S, Abe H, Okano HJ, Hara-Miyauchi C, Okano H, et al Host-derived MMP-13 exhibits a protective role in lung metastasis of melanoma cells by local endostatin production. Br J Cancer. 2011;105: 1615–1624. 10.1038/bjc.2011.431 22015555 PMC3242531

[25] Rodriguez A, Hilvo M, Kytomaki L, Fleming RE, Britton RS, Bacon BR, et al Effects of iron loading on muscle: genome-wide mRNA expression profiling in the mouse. BMC Genomics. 2007;8: 379 17949489 10.1186/1471-2164-8-379 PMC2151772

[26] Sandler NG, Mentink-Kane MM, Cheever AW, Wynn TA Global gene expression profiles during acute pathogen-induced pulmonary inflammation reveal divergent roles for Th1 and Th2 responses in tissue repair. J Immunol. 2003;171: 3655–3667. 14500663 10.4049/jimmunol.171.7.3655

[27] Amante FH, Stanley AC, Randall LM, Zhou Y, Haque A, McSweeney K, et al A role for natural regulatory T cells in the pathogenesis of experimental cerebral malaria. Am J Pathol. 2007;171: 548–559. 17600128 10.2353/ajpath.2007.061033 PMC1934517

[28] Burke ML, McManus DP, Ramm GA, Duke M, Li Y, Jones MK, et al Co-ordinated gene expression in the liver and spleen during *Schistosoma japonicum* infection regulates cell migration. PLoS Negl Trop Dis. 2010;4: e686 10.1371/journal.pntd.0000686 20502518 PMC2872641

[29] Stoff-Khalili MA, Rivera AA, Le LP, Stoff A, Everts M, Contreras JL, et al Employment of liver tissue slice analysis to assay hepatotoxicity linked to replicative and nonreplicative adenoviral agents. Cancer Gene Ther. 2006;13: 606–618. 16410819 10.1038/sj.cgt.7700934

[30] Guyot C, Lepreux S, Combe C, Sarrazy V, Billet F, Balabaud C, et al Fibrogenic cell phenotype modifications during remodelling of normal and pathological human liver in cultured slices. Liver Int. 2010;30: 1529–1540. 10.1111/j.1478-3231.2010.02342.x 20846345

[31] Baldim LB, Nejo R Jr, Souza ME, Gomes MC, Picinato MA, Fina CF, et al Effect of hyperbaric oxygen therapy on liver function during intermittent ischemia. Acta Cir Bras. 2013;28 Suppl 1: 61–65. 23381826 10.1590/s0102-86502013001300012

[32] Sasaki M, Yoneda N, Kitamura S, Sato Y, Nakanuma Y A serum amyloid A-positive hepatocellular neoplasm arising in alcoholic cirrhosis: a previously unrecognized type of inflammatory hepatocellular tumor. Mod Pathol. 2012;25: 1584–1593. 10.1038/modpathol.2012.114 22766792

[33] Hansen MT, Forst B, Cremers N, Quagliata L, Ambartsumian N, Grum-Schwensen B, et al A link between inflammation and metastasis: serum amyloid A1 and A3 induce metastasis, and are targets of metastasis-inducing S100A4. Oncogene. 2014. 10.1038/onc.2013.568 24469032

[34] Ren SW, Qi X, Jia CK, Wang YQ Serum amyloid A and pairing formyl peptide receptor 2 are expressed in corneas and involved in inflammation-mediated neovascularization. Int J Ophthalmol. 2014;7: 187–193. 10.3980/j.issn.2222-3959.2014.02.01 24790856 PMC4003068

[35] Niemi K, Teirila L, Lappalainen J, Rajamaki K, Baumann MH, Oorni K, et al Serum amyloid A activates the NLRP3 inflammasome via P2X7 receptor and a cathepsin B-sensitive pathway. J Immunol. 2011;186: 6119–6128. 10.4049/jimmunol.1002843 21508263

[36] Moles A, Murphy L, Wilson CL, Chakraborty JB, Fox C, Park EJ, et al A TLR2/S100A9/CXCL-2 signaling network is necessary for neutrophil recruitment in acute and chronic liver injury in the mouse. J Hepatol. 2014;60: 782–791. 10.1016/j.jhep.2013.12.005 24333183 PMC3960359

[37] Kuboki S, Shin T, Huber N, Eismann T, Galloway E, Schuster R, et al Hepatocyte signaling through CXC chemokine receptor-2 is detrimental to liver recovery after ischemia/reperfusion in mice. Hepatology. 2008;48: 1213–1223. 10.1002/hep.22471 18688883 PMC2695827

[38] Chuah C, Jones MK, Burke ML, McManus DP, Owen HC, Gobert GN Defining a pro-inflammatory neutrophil phenotype in response to schistosome eggs. Cell Microbiol. 2014. 10.1111/cmi.12316 24898449

[39] McDonald B, Pittman K, Menezes GB, Hirota SA, Slaba I, Waterhouse CC, et al Intravascular danger signals guide neutrophils to sites of sterile inflammation. Science. 2010;330: 362–366. 10.1126/science.1195491 20947763

[40] Hickey MJ, Westhorpe CL Imaging inflammatory leukocyte recruitment in kidney, lung and liver—challenges to the multi-step paradigm. Immunol Cell Biol. 2013;91: 281–289. 10.1038/icb.2012.83 23337698

[41] Tremble P, Chiquet-Ehrismann R, Werb Z The extracellular matrix ligands fibronectin and tenascin collaborate in regulating collagenase gene expression in fibroblasts. Mol Biol Cell. 1994;5: 439–453. 7519905 10.1091/mbc.5.4.439 PMC301053

[42] Tamai K, Fukushima K, Ueno Y, Moritoki Y, Yamagiwa Y, Kanno N, et al Differential expressions of aquaporin proteins in human cholestatic liver diseases. Hepatol Res. 2006;34: 99–103. 16406793 10.1016/j.hepres.2005.11.006

[43] Mahmoud KM, Sobh MA, El-Agroudy AE, Mostafa FE, Baz ME, Shokeir AA, et al Impact of schistosomiasis on patient and graft outcome after renal transplantation: 10 years' follow-up. Nephrol Dial Transplant. 2001;16: 2214–2221. 11682670 10.1093/ndt/16.11.2214

[44] Sakurai T, He G, Matsuzawa A, Yu GY, Maeda S, Hardiman G, et al Hepatocyte necrosis induced by oxidative stress and IL-1 alpha release mediate carcinogen-induced compensatory proliferation and liver tumorigenesis. Cancer Cell. 2008;14: 156–165. 10.1016/j.ccr.2008.06.016 18691550 PMC2707922

[45] Seki E, De Minicis S, Gwak GY, Kluwe J, Inokuchi S, Bursill CA, et al CCR1 and CCR5 promote hepatic fibrosis in mice. J Clin Invest. 2009;119: 1858–1870. 19603542 10.1172/JCI37444 PMC2701864

[46] Seki E, De Minicis S, Osterreicher CH, Kluwe J, Osawa Y, Brenner DA, et al TLR4 enhances TGF-beta signaling and hepatic fibrosis. Nat Med. 2007;13: 1324–1332. 17952090 10.1038/nm1663

[47] Lee SC, Brummet ME, Shahabuddin S, Woodworth TG, Georas SN, Leiferman KM, et al Cutaneous injection of human subjects with macrophage inflammatory protein-1 alpha induces significant recruitment of neutrophils and monocytes. J Immunol. 2000;164: 3392–3401. 10706735 10.4049/jimmunol.164.6.3392

[48] Jain A, Kaczanowska S, Davila E IL-1 Receptor-Associated Kinase Signaling and Its Role in Inflammation, Cancer Progression, and Therapy Resistance. Front Immunol. 2014;5: 553 10.3389/fimmu.2014.00553 25452754 PMC4233944

[49] Seki E, Tsutsui H, Nakano H, Tsuji N, Hoshino K, Adachi O, et al Lipopolysaccharide-induced IL-18 secretion from murine Kupffer cells independently of myeloid differentiation factor 88 that is critically involved in induction of production of IL-12 and IL-1beta. J Immunol. 2001;166: 2651–2657. 11160328 10.4049/jimmunol.166.4.2651

[50] Thobe BM, Frink M, Hildebrand F, Schwacha MG, Hubbard WJ, Choudhry MA, et al The role of MAPK in Kupffer cell toll-like receptor (TLR) 2-, TLR4-, and TLR9-mediated signaling following trauma-hemorrhage. J Cell Physiol. 2007;210: 667–675. 17117477 10.1002/jcp.20860

[51] Matsuno K, Nomiyama H, Yoneyama H, Uwatoku R Kupffer cell-mediated recruitment of dendritic cells to the liver crucial for a host defense. Dev Immunol. 2002;9: 143–149. 12885155 10.1080/1044667031000137610 PMC2276107

[52] Fabre V, Wu H, PondTor S, Coutinho H, Acosta L, Jiz M, et al Tissue inhibitor of matrix-metalloprotease-1 predicts risk of hepatic fibrosis in human *Schistosoma japonicum* infection. J Infect Dis. 2011;203: 707–714. 10.1093/infdis/jiq099 21199883 PMC3072733

[53] McDonald EA, Friedman JF, Sharma S, Acosta L, Pond-Tor S, Cheng L, et al *Schistosoma japonicum* soluble egg antigens attenuate invasion in a first trimester human placental trophoblast model. PLoS Negl Trop Dis. 2013;7: e2253 10.1371/journal.pntd.0002253 23755313 PMC3675010

[54] McDonald EA, Cheng L, Jarilla B, Sagliba MJ, Gonzal A, Amoylen AJ, et al Maternal infection with *Schistosoma japonicum* induces a profibrotic response in neonates. Infect Immun. 2014;82: 350–355. 10.1128/IAI.01060-13 24166958 PMC3911825

